# Phase Equilibrium of *n*-Nonane + *n*-Decane for Low-Temperature Thermal Energy Storage: Insights into Odd–Even Effects

**DOI:** 10.1007/s10765-025-03531-7

**Published:** 2025-03-06

**Authors:** Maria C. M. Sequeira, Timur Nikitin, Fernando J. P. Caetano, Hermínio P. Diogo, João M. N. A. Fareleira, Rui Fausto

**Affiliations:** 1 Departamento de Engenharia Química, Centro de Química Estrutural, Institute of Molecular Sciences, Instituto Superior Técnico, Universidade de Lisboa, Av. Rovisco Pais, 1049-001 Lisbon, Portugal; 2 Departamento de Química, CQC-IMS, Universidade de Coimbra, Rua Larga, 3004-535 Coimbra, Portugal; 3 Departamento de Ciências e Tecnologia, Universidade Aberta, Rua da Escola Politécnica, 147, 1269-001 Lisbon, Portugal; 4 Centro de Química Estrutural, Institute of Molecular Sciences, Instituto Superior Técnico, Universidade de Lisboa, Av. Rovisco Pais, 1049-001 Lisbon, Portugal; 5 Department of Physics, Faculty of Sciences and Letters, ERA-Chair Spectroscopy@IKU, Istanbul Kultur University, Ataköy Campus, Bakirköy, 34156 Istanbul, Turkey

**Keywords:** Low-temperature thermal energy storage (TES), *n*-alkanes, Odd–even effects, Phase change material (PCM), Solid–liquid phase diagram

## Abstract

The present article presents the solid–liquid phase diagram of a binary system composed of an *n*-alkane with an odd number of carbon atoms, namely, *n*-nonane (*n*-C_9_), with an even-numbered one, namely *n*-decane (*n*-C_10_). This work is part of a series of phase equilibrium studies on *n*-alkanes for low-temperature thermal energy storage (TES) applications. The ultimate purpose of this work is to investigate the adequacy of this binary system to be used as a Phase Change Material (PCM) at low temperatures. Additionally, along the equilibrium studies on linear alkanes, an interesting feature has emerged: the solid–liquid phase diagrams of binary *n*-alkane systems apparently show a striking dependence on the odd or even number of carbon atoms in their chains. The phase diagram for the system *n*-C_9_ + *n*-C_10_ has primarily been obtained using Differential Scanning Calorimetry (DSC), whose results have been complemented by Hot-Stage Microscopy (HSM) and low-temperature Raman Spectroscopy results. The DSC analysis provided both the temperature and enthalpy values for the observed solid–liquid and solid–solid phase transitions. The *n*-C_9_ + *n*-C_10_ binary system seems to display a peritectic solid–liquid phase diagram at low temperatures. The peritectic temperature found was 222.41 K, with a peritectic composition around *x*_nonane_ = 0.60. Those results confirmed the initial suggestions that this would be a peritectic system, based on previously observed odd–even effects on phase equilibrium behavior of alkane mixtures. The goal of this work is to extend new insights into the solid–liquid phase equilibrium behavior of the binary system *n*-C_9_ + *n*-C_10_, a topic not yet covered in the literature. This information, consequently, provides practical and essential information on the potential use of this system as PCM for low-temperature TES applications. Additionally, it contributes to support the important discussion on the effect of odd–even number of carbons of the individual *n*-alkanes in the solid–liquid phase equilibrium behavior of their binary mixtures. The solid–liquid diagram of this system is being published for the first time, as far as the authors are aware.

**Supplementary Information:**

The online version contains supplementary material available at 10.1007/s10765-025-03531-7.

## Introduction

Thermal Energy Storage (TES) is a crucial technology for tackling the challenges raised by the variability and intermittency of renewable energy sources. By storing surplus energy when production exceeds demand and releasing it during periods of scarcity, TES helps to stabilize the energy supply, to make it more consistent and reliable. This is particularly relevant in the industrial area, where latent heat technologies represent a compact and practical solution for dealing with the gap between energy demand and supply.

Over the years, *n*-alkanes have been widely studied for different applications in many diverse areas. Recently, they became even more popular in the energy field because of their exceptional characteristics as PCM for thermal energy storage (TES) applications. The importance of energy storage, in particular, latent heat thermal energy storage, has increased significantly in recent years. A major factor driving this growth is the need to store excess heat generated in industrial processes and to address the intermittent nature of renewable energy sources [1, 2].

Recently, our group has developed studies on the solid–liquid phase equilibrium of binary systems with potential application as phase change materials (PCM) at low temperatures. That study has involved two di-*n*-alkyl adipates [3] and three different binary alkane systems [2, 4].

Our previous studies on *n*-alkane systems have evidenced some interesting characteristics of the *n*-alkanes family, relating the odd or even number of carbon atoms of the individual molecules to the type of their binary solid–liquid phase diagrams. The influence of the even or odd number of carbon atoms in the *n*-alkane chain, or the presence of substituent groups in non-linear alkyl chain molecules, has been extensively documented in relation to various thermophysical properties in the solid state. These include melting points, enthalpies of fusion, enthalpies of sublimation, and density [5–7]. The corresponding effects on the solid–liquid phase diagrams for *n*-alkane binary systems, have been reported by Espeau [8] and Mondieig [9] and have also been addressed in the review by Gunasekara et al. [10]. From the works of those authors some tendencies relating the main characteristics of the phase diagrams with the even and/or odd number of carbon atoms in the *n*-alkane chains have been identified.

Numerous studies have been conducted over the years by various research groups to investigate the solid-phase properties of *n*-alkanes and compounds containing alkyl groups, with the aim of better understanding the underlying phenomena in these materials [7–9, 11]. It has been shown that *n*-alkanes exhibit different crystal packing arrangements, according to their odd or even number of carbon atoms in the chain. As a result, several properties, particularly those associated with the solid-state characteristics, are found to be influenced by whether the carbon chain contains an odd or even number of atoms.

Recently, a brief review [2] has been reported focusing on the polymorphism features of pure *n*-alkanes previously described in the literature [8–11]. As mentioned before by Sequeira et al. [2], those effects may be used as an advantage for some specific applications of alkane systems, for example, as candidates to be PCM for low-temperature TES. In fact, it is of utmost importance to be able to predict the solid–liquid type of diagrams of those systems, having in view the selection of alkane mixtures for application as PCM.

The solid–liquid phase diagram types for binary mixtures of *n*-alkanes that can be found in the literature vary from eutectics and congruent melting solid solutions to peritectics [9, 10]. According to Gunasekara et al. [10], the PCM ideal phase change characteristics for blends can be found primarily in congruent melting systems (solid solutions or compounds) and in eutectics. It can be noted that, although peritectic systems exhibit less favorable characteristics (incomplete phase transitions, metastability, and phase separation), they may still hold some potential significance for use as PCM [10]. It is important to stress that, although the solid–liquid phase diagrams of binary *n*-alkane mixtures may indicate a tendency to be organized according to the odd or even number of carbon atoms of the individual n-alkanes, numerous exceptions to this pattern are documented in the literature.

Our previous studies on three different binary *n*-alkane systems have evidenced two different types of phase diagrams. In particular, the systems *n*-octane + *n*-decane (*n*-C_8_ + *n*-C_10_) and *n*-decane + *n*-dodecane (*n*-C_10_ + *n*-C_12_) [4] have both shown to have eutectic phase diagrams, and the system *n*-nonane + *n*-undecane (*n*-C_9_ + *n*-C_11_) [2] have evidenced to possess a congruent melting solid solution. Those works confirmed, for short chain *n*-alkane mixtures, the tendency for even–even systems to give rise to eutectic phase diagrams and odd–odd systems to originate congruent melting solid solutions.

Solid–liquid phase diagrams provide critical perceptions into the melting and solidification behavior of binary mixtures. These diagrams assign essential insights into the interactions between components and their phase transitions, which are vital for optimizing TES systems and enhancing the performance of PCM. The construction of the solid–liquid phase diagram for the system under study, *n*-nonane (*n*-C_9_) and *n*-decane (*n*-C_10_), combines the use of Differential Scanning Calorimetry (DSC), Hot-Stage Microscopy (HSM), and Raman spectroscopy, allowing a comprehensive understanding of the phase behavior of the binary system. Those complementary data are essential for completing our phase equilibrium studies on *n*-alkane mixtures at sub-zero temperatures, an area where available data are particularly scarce. The present integrated approach ensures an accurate construction of the solid–liquid phase diagram, offering valuable insights for TES applications at low temperatures.

A particularly important property for PCM applications, examined in the present work, is the enthalpy of the solid–liquid phase transition, which is essential to be addressed in this type of study.

The new *n*-alkane system dealt with in the present article, involves an odd and an even number of carbon atoms of the pure compounds, which, according to previous works [2, 4, 9, 10], can be expected to be a peritectic system. The present study allowed to obtain a comprehensive view of the phase equilibrium behavior of this binary system, confirming the previous suggestion.

## Experimental

### Materials

In this work, *n*-C_9_ and *n*-C_10_ were used as received. The water content of the used materials was measured using a Karl Fischer 831 KF Coulometer from Metrohm. Table 1 shows the characterization of the material samples used in this work.

**Table 1:** Characterization of the liquids used in this work

Name	CAS number	Supplier	Lot Number	Water content (mg·kg^−1^)	Purity (mass fraction) (%)
*n*-nonane	111-65-9	Thermo Scientific	10228094	41.5	99.3
*n*-decane	124-18-5	TCI Chemicals	OL5LO-XZ	19.8	100

Purity as stated in the corresponding analysis certificate and water content as measured in situ

In view of the high purity of the compounds available in the certificates of analysis provided by the suppliers for the batches in use and the water contents level established immediately before the experimental measurements, the samples were used as received. To achieve the highest accuracy in composition, the binary mixtures for this investigation were prepared gravimetrically using a Mettler Toledo MS205DU micro-balance with a precision of ± 0.01 mg.

### Techniques

#### Differential Scanning Calorimetry (DSC)

The calorimetric response of different samples was measured using a differential scanning calorimeter 2920 MDSC system from TA Instruments Inc. The experimental procedure is described in detail in references [2–4]. Therefore, a summary description is presented here. The sample masses of 4.0–6.0 mg were sealed in air inside aluminum pans, weighed with a precision of ± 1.0 × 10^–4^ mg using a Mettler UMT2 ultra-micro-balance and subsequently, analyzed by differential scanning calorimetry (DSC) at a scanning rate, *β* = 5 K·min^–1^. Helium (Air Liquide N55), at a flow rate of 30 cm^3^·min^−1^ was used as purging gas. The baseline was corrected by performing a scan with an empty pan over the temperature range of the experiments. The temperature and heat flow scales of the instrument were calibrated at different heating rates, based on the onsets of the fusion peaks of high-purity standards. Features of the calibration procedure are explained elsewhere [12].

#### Hot-Stage Microscopy (HSM)

Polarized optical microscopy observations were performed on an Olympus BX51 optical microscope. The temperature changes and stabilization were set by a Linkam LTS360 liquid nitrogen cooled cryostage and was measured with a Pt resistance thermometer. The liquid samples were first placed on the glass plate, covered by a second glass and cooled down at 15 K·min^−1^. After solidification, the microstructure of the sample was monitored with an Olympus C5060 wide zoom camera for picture and/or movie record. Images were recorded with 250 × magnification in the temperature range from 173.15 K to 293.15 K at a heat rate of 10 K·min^−1^.

#### Raman Spectroscopy

The Raman spectra were acquired with a Horiba LabRam HR Evolution micro-Raman system using a solid-state laser (λ = 532 nm, ~ 5 mW on the sample) for excitation. The samples were probed with a 50 × objective, and the laser spot diameter on the sample was approximately 1 μm. The system was calibrated using a Si crystal as a reference (reference band at 520.5 cm^–1^). The presented spectra were typically acquired with an acquisition time of 5 s and averaged over 10 spectra accumulations. The spectral resolution is about 5 cm^–1^. The temperature variation measurements with an accuracy of about 0.01 K were carried out using the following Linkam Scientific instruments: THMS 600 stage and an LNP95 cooling system controlled by a T95-PE Linkpad controlling unit.

To evaluate the thermal behavior and evolution of the mixtures, especially for the phase transitions, the samples were cooled until the complete solidification at 193.15 K at a cooling rate of 10 K·min^−1^. The samples were then heated at a rate of 5 K·min^−1^ (to be comparable with the DSC results) until melting, and Raman spectra and optical images were collected at various temperatures. The spectra were also obtained at room temperature before cooling the samples.

## Results and Discussion

Our previous work [2, 4] and several other literature studies [9, 10] have shown the complexity of the solid–liquid equilibria of *n*-alkanes, both for pure compounds and their binary mixtures. The system *n*-C_9_ and *n*-C_10_ under study is not an exception, since polymorphic transitions are observed which slightly complicate the interpretation of phase equilibrium behavior.

As in our previous studies, three different techniques were used to correctly depict the phase equilibrium characteristics of the present binary system: DSC, HSM, and variable temperature Raman spectroscopy.

### Differential Scanning Calorimetry (DSC)

**Fig. 1:**
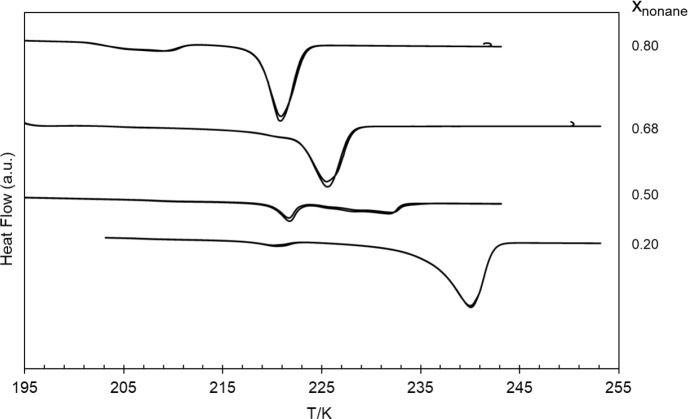
DSC heating curves of some selected binary mixtures, with compositions indicated by the *x*_nonane_ molar fraction. The scanning rate was *β* = 5 K·min^–1^ (exo up). Two consecutive heating runs are presented for each composition

DSC heating curves for the most relevant binary mixtures are shown in Fig. 1. The results for pure compounds, *n*-C_9_ and *n*-C_10_, have already been provided in our previous articles [2, 4].

The reported values for the temperature and the enthalpy of fusion, given in Table 2, were obtained by averaging the values from two consecutive cycles. The DSC results, heating curves, temperature, and enthalpy of fusion, for all remaining studied compositions are presented in section S1 of the Supplementary Information (Fig. S1 and Table S1). The experimental *solidus* and *liquidus* temperatures in Fig. 12 (phase diagram) were obtained from the endothermic peaks detected in thermograms.

**Table 2:** DSC data for pure *n-*C_9_ [2], pure *n*-C_10_ [4], and three of their binary mixtures, including the onset temperatures, *T*_*onset*_, maximum peak temperatures, *T*_*max*_, and the corresponding enthalpies of fusion, Δ_fus_*H*, at atmospheric pressure, 0.1 MPa. The scanning rate was *β* = 2 K·min^–1^ for pure *n-*C_9_ and *β* = 5 K·min^–1^ for *n-*C_10_ and for the binary mixtures [2, 4]

*x*_nonane_	DSC 1st peak			DSC 2nd peak		
	T_onset_ /K	T_max_ /K	Δ_fus_***H*** /J·g^–1^	T_onset_ /K	T_max_ /K	Δ_fus_***H*** /J·g^–1^
1	216.46	217.12	3.8	218.28	219.34	7.4
0.80	201.23	208.60	22.0	218.53	220.83	104.7
0.68	218.48	222.42	116.3	–	–	–
0.50	219.81	221.67	38.7	224.67	231.95	54.7
0.20	217.14	220.11	6.2	235.51	240.03	146.1
0	240.19	242.93	169.4	–	–	–

Expanded uncertainties for a 95% confidence level (*k* = 2): *U*(*x*) = 0.00007; *U*(*T*) = 0.25 K; *U*(Δ_fus_*H)* = 8.1 J·g^–1^ (see Supp. Information – S4)

Figure 1 depicts different thermograms according to the molar composition, *x*_nonane_, of the samples. In what regards binary mixtures, it is possible to depict different behaviors with some mixtures presenting two peaks and others only one peak. Further analysis of the binary mixtures using the results of HSM and Raman spectroscopy to complete these DSC results have enabled to draw some important conclusions, which are presented in Sects. 3.2 and 3.3.

### Hot-Stage Microscopy (HSM)

**Fig. 2:**
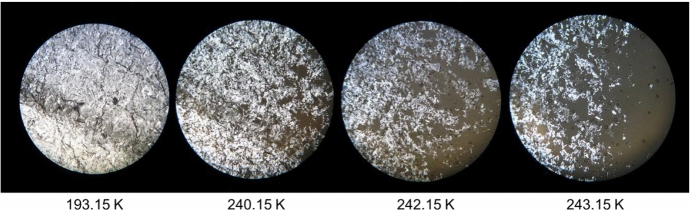
HSM images of *n*-C_10_ acquired upon heating the sample in the temperature range from 193.15 K to 243.15 K, employing a magnification of 250 ×

**Fig. 3:**
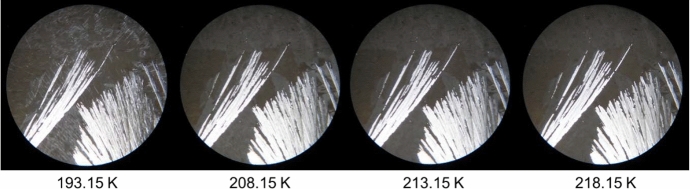
HSM images of the binary mixture with molar fraction *x*_nonane_ = 0.80 acquired upon heating in the temperature range from 193.15 K to 218.15 K, employing a magnification of 250 ×

**Fig. 4:**
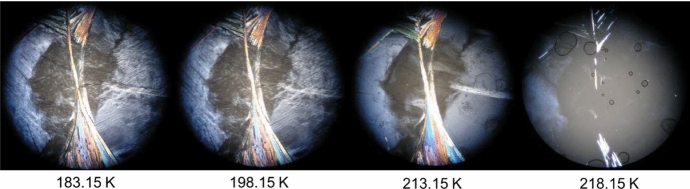
HSM images of the binary mixture with molar fraction *x*_nonane_ = 0.68 acquired upon heating in the temperature range from 183.15 K to 218.15 K, employing a magnification of 250 ×

**Fig. 5:**
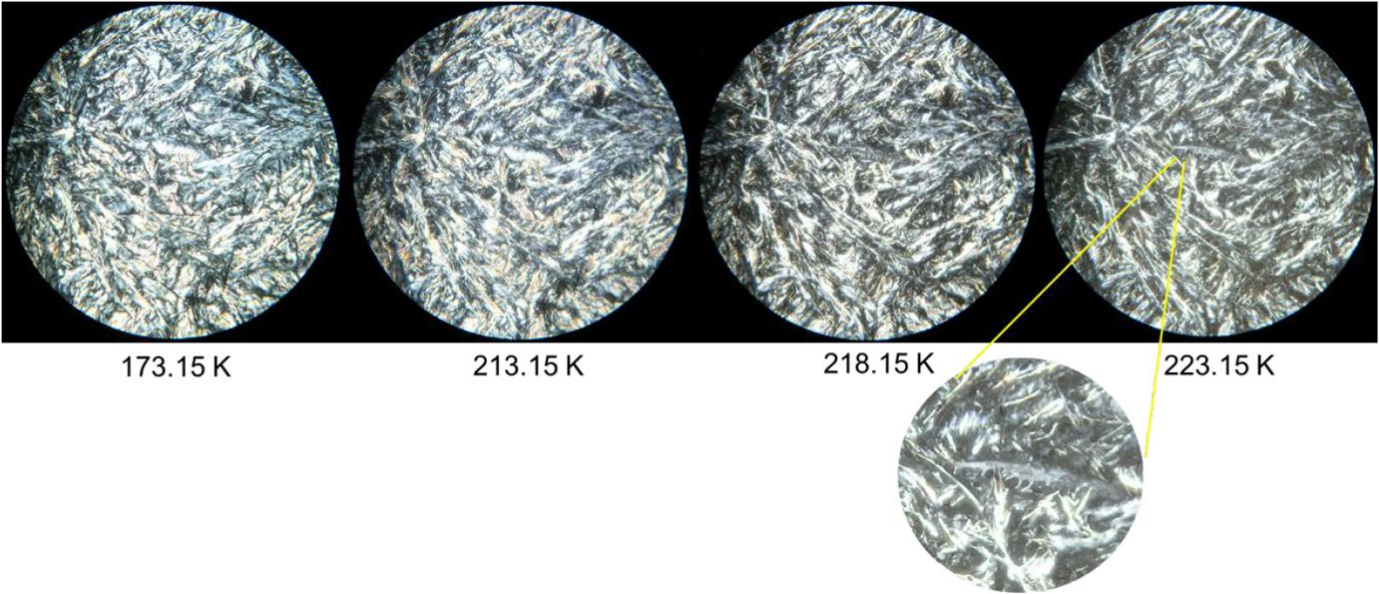
HSM images of the binary mixture with molar fraction *x*_nonane_ = 0.50 acquired upon heating in the temperature range from 173.15 K to 223.15 K, employing a magnification of 250 ×. The zoom applied to the last image shows the drops of liquid already formed in the sample

**Fig. 6:**
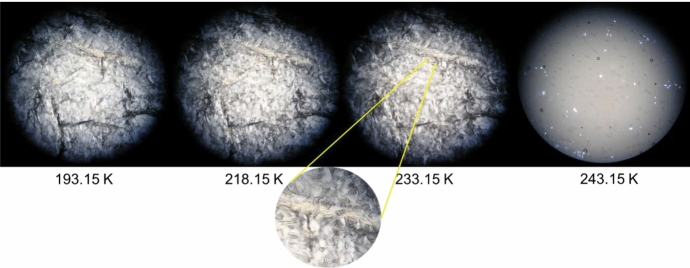
HSM images of the binary mixture with molar fraction *x*_nonane_ = 0.20 acquired upon heating in the temperature range from 193.15 K to 243.15 K, employing a magnification of 250 ×. The zoom applied to the image at 233.15 K shows the drops of liquid already formed in the sample

Hot-Stage Microscopy is an effective technique for observation of thermal transitions in real time, thus providing valuable information about the thermal behavior and stability of a given material, including the identification of polymorphic transitions [13]. This feature has been evidenced in our previous works [2, 3], where results from DSC measurements were confirmed and/or complemented by HSM data. Using light polarization, it is possible to identify both solid–liquid and solid–solid transitions. In the present work, this technique has once again been used and again proved its effectiveness. The obtained HSM images for the pure compound *n*-C_10_ and the most relevant binary mixtures are presented in Figs. 2, 3, 4, 5, and 6. In section S2 of the Supplementary Information, it is possible to find HSM images for the remaining binary mixtures, including the ones for pure *n*-C_9_ that were previously published in our recent article [2].

Figure 2 displays the HSM images for pure *n*-C_10_ as a function of the temperature. In the studied interval of temperature, this compound does not show any polymorphic transition, and so, it is only possible to observe the solid–liquid transition, which starts at 240.15 K and continues to evolve up to approximately 243.15 K.

Figure 3 shows the HSM images for the binary mixture *x*_nonane_ = 0.80 with the corresponding temperatures between 193.15 K and 218.15 K. At 193.15 K, it is possible to identify a fully solid sample that evolves gradually from 208.15 K to 218.15 K to another image, as the DSC results have shown before. The change in the observed texture in the images presented in Fig. 3, without any alteration in light polarization, suggests the presence of polymorphism. At 223.15 K, the mixture was already in the liquid phase, which also corroborates the results obtained from DSC.

For the binary mixture *x*_nonane_ = 0.68, the HSM results are shown in Fig. 4. For this sample, the solid phase is clearly visible at 183.15 K, and it is maintained until 218.15 K. At 213.15 K, a subtle change is visible, which is attributed to a polymorphic transition that is barely detected by DSC. Almost simultaneously, the solid–liquid transition begins at 218.15 K and occurs quite rapidly. Consequently, at 218.15 K, the sample is mainly in the liquid state, as it is possible to observe in the pictures in Fig. 4.

The HSM images for the binary mixture *x*_nonane_ = 0.50 are presented in Fig. 5. The solid phase is observed between 173.15 K and 218.15 K, when the first drops of liquid started to appear. At 213.15 K, it was possible to observe a subtle change in the color of the sample, which is not very perceptible in the shown image that seems to indicate a polymorphic transition. This subtle change is also compatible with the DSC experiments, where only a small perturbation of the baseline was observed. At 223. 15 K, the melting continues to evolve, and at 228.15 K the mixture was already an isotropic liquid.

Finally, the HSM results for the binary mixture *x*_nonane_ = 0.20 are shown in Fig. 6. Here, the solid state is visible at 193.15 K. At 218.15 K, the polymorphic transition is quite difficult to detect, but still possible with the subtle color modification observed better in the bottom part of the image. At 233.15 K, the sample begins to show small drops of liquid (zoom in Fig. 6), and at 243.15 K, it is in the liquid state, in agreement with the DSC results.

### Raman Spectroscopy

Raman spectroscopy offers crucial insights for the construction of the binary phase diagram, shedding light on both phase transitions solid–solid and solid–liquid and the solid phase itself. In the context of phase transitions, this technique is particularly useful in detecting polymorphic events enabling the identification of the DSC peaks and the assignment of these peaks to S–L or S–S transitions. Additionally, it is especially important to understand the solid phase of each system, since it enables to distinguish between a solid solution, where the solid is the mixture of the two compounds (isomorphism), and a mixture of two solids, where each solid is one pure compound (non-isomorphism). This information is extremely useful for the characterization of a binary phase diagram and, in particular, for TES applications, which utilize the solid–liquid phase transition to store energy. In fact, this approach has allowed our group to identify different types of systems in previous studies [2–4].

The results of Raman spectroscopy for the pure compound *n*-C_9_ have been previously published by our group [2]. Thus, only the marker bands for this compound will be presented here in order to facilitate the reading and interpretation of the results. The Raman spectra previously obtained for this compound [2] are presented in Fig. S8 in the Supplementary Information.

**Fig. 7:**
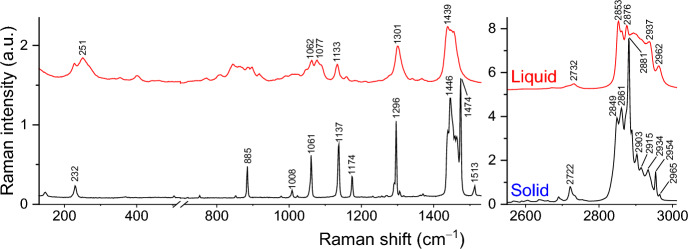
Raman spectra of solid and liquid phase samples of *n*-C_10_

The results for the pure compound *n*-C_10_ have also been published before by our group [4]; however, new experiments were conducted specifically for the present study, in order to compare the results for both pure compounds under the same conditions. The Raman spectra obtained for both solid and liquid phases of pure *n*-C_10_ are presented in Fig. 7.

**Fig. 8:**
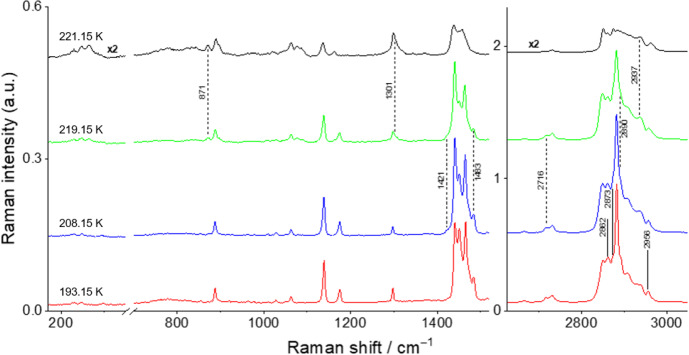
Temperature variation Raman spectra for the binary mixture *x*_*nonane*_ = 0.80

**Fig. 9:**
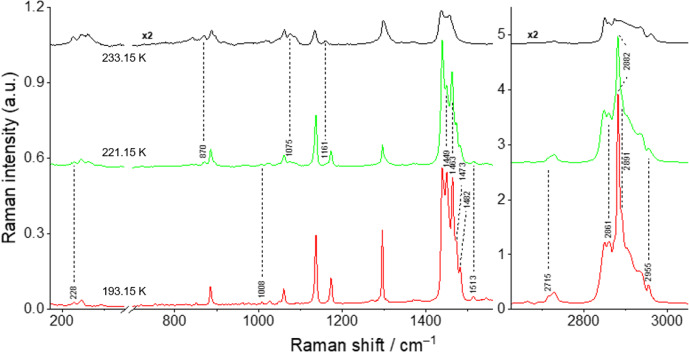
Temperature variation Raman spectra for the binary mixture *x*_nonane_ = 0.68

**Fig. 10:**
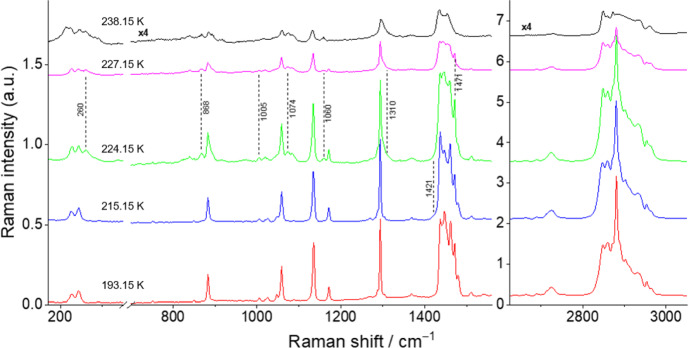
Temperature variation Raman spectra for the binary mixture *x*_nonane_ = 0.50

**Fig. 11:**
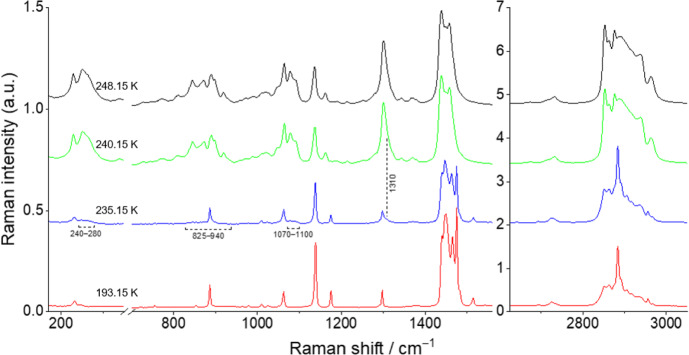
Temperature variation Raman spectra for the binary mixture *x*_nonane_ = 0.20

The spectra for the binary mixtures *x*_nonane_ = 0.80, 0.68, 0.50, and 0.20 at different temperatures are shown in Figs. 8, 9, 10, and 11 and are discussed here, once they represent the three different types of behavior found for the studied binary mixtures. The Raman spectra obtained for the remaining studied binary compositions are presented in the Supplementary Information (section S3, Figs. S8–S12).

For the two pure compounds of the studied binary system (*n*-C_9_, *n*-C_10_), Raman spectroscopy, as expected, detects polymorphism only for *n*-C_9_. The pure compound *n*-C_10_ does not evidence polymorphism. This phenomenon, first observed in the DSC experiments, was then fully confirmed by the Raman experiments.

The marker bands for both pure compounds in their solid and liquid phases are as follows:

**n** **-C**_**9**_

- Solid T_i_ (193.15 K): 247 cm^–1^, 293 cm^–1^, 885 cm^–1^, 1048 cm^–1^, 1173 cm^–1^, 1295 cm^–1^, 1438 cm^–1^, 1448 cm^–1^, 1464 cm^–1^, 1483 cm^–1^, 1545 cm^–1^, 2713 cm^–1^, 2869 cm^–1^, 2879 cm^–1^, 2889 cm^–1^, 2904 cm^–1^, 2950 cm^–1^, and 2962 cm^–1^.
- Solid R_I_ (218.15 K): 247 cm^–1^, 885 cm^–1^, 1048 cm^–1^, 1173 cm^–1^, 1295 cm^–1^, 1420(sh.) cm^–1^, 1438 cm^–1^, 1459 cm^–1^, 2712 cm^–1^, 2843 cm^–1^, 2870(sh.) cm^–1^, 2877, 2887(sh.) 2904 cm^–1^, and 2954 cm^–1^.
- Liquid (220.15 K): 247 cm^–1^, 264 cm^–1^, 870 cm^–1^, 889 cm^–1^, 1019 cm^–1^, 1076 cm^–1^, 1160 cm^–1^, 1300 cm^–1^, 1440 cm^–1^, 1455 cm^–1^, 2711(sh.) cm^–1^, 2728 cm^–1^, 2850 cm^–1^, 2860 cm^–1^, 2873 cm^–1^, 2935 cm^–1^, and 2960 cm^–1^ (Narrow Band! a band at 215 cm^–1^ appears that is not present at RT).

**n-C**_**10**_

- Solid Phase (193.15 K): 147 cm^–1^, 230 cm^–1^, 504 cm^–1^, 885 cm^–1^, 1008 cm^–1^, 1061 cm^–1^, 1137 cm^–1^, 1174 cm^–1^, 1296 cm^–1^, 1446 cm^–1^, 1474 cm^–1^, 1513 cm^–1^, 2722 cm^–1^, 2849 cm^–1^, 2861 cm^–1^, 2873(sh.) cm^–1^, 2881 cm^–1^, 2903 cm^–1^, 2915 cm^–1^, 2934 cm^–1^, 2954 cm^–1^, and 2965 cm^–1^.
- Liquid Phase (298.15 K): 251, 401, a group at 750–950 cm^–1^, 1062 cm^–1^, 1077 cm^–1^, 1133 cm^–1^, 1301 cm^–1^, 1439 cm^–1^, 2732 cm^–1^, 2853 cm^–1^, 2876 cm^–1^, 2937 cm^–1^, and 2962 cm^–1^.

For the *x*_nonane_ = 0.80 sample (Fig. 8), at 193.15 K, it is possible to identify all the bands in the fingerprint region corresponding to the *n*-C_9_ T_i_ solid phase. In the CH stretching region, the spectrum of the mixture is more distinct from that of the pure *n*-C_9_ T_i_ solid phase. This region is indeed more similar to that of the *n*-C_10_ solid phase, exhibiting features at 2862, 2873 (sh.), and 2956 cm^–1^. At 208.15 K, a weak broad band at 1421 cm^–1^ appears, coinciding with a similar feature found in the spectrum of the *n*-C_9_ R_I_ solid phase, which could indicate the beginning of the polymorphic transition. Although no other spectral similarities with the *n*-C_9_ R_I_ solid phase have been observed to confirm this process, the potential onset of the polymorphic transition at 208.15 K aligns with the DSC results, which show a peak at about this temperature. The polymorphic transition continues to progress until 219.15 K, where the spectrum displays marker bands of the liquid phases at 871 cm^–1^, 1301 cm^–1^, and 2937 cm^–1^ for both *n*-C_9_ and *n*-C_10_. At this temperature, the marker band at 1421 cm^–1^, corresponding to the *n*-C_9_ R_I_ solid phase, disappears. However, some peaks for the *n*-C_9_ T_i_ solid phase remain, specifically at 1483 cm^–1^, 2716 cm^–1^, and 2890 cm^–1^. At 221.15 K, the spectrum shows that the sample is completely in the liquid phase, with the marker bands at 265 cm^–1^, 871 cm^–1^, 889 cm^–1^, 1020 cm^–1^, 1076 cm^–1^, 1162 cm^–1^, 2938 cm^–1^, 2874 cm^–1^, and 2960 cm^–1^ for the *n*-C_9_ and *n*-C_10_ liquid phases. Additionally, the peaks corresponding to the *n*-C_9_ T_i_ solid phase that were still present at 219.15 K have disappeared. The spectrum at 221.15 K is comparable to the one obtained at room temperature.

The sample *x*_nonane_ = 0.68 (Fig. 9) at 193.15 K presents a spectrum that is a superposition of those of its components, *n*-C_9_ T_i_ and *n*-C_10_ solid phases. More specifically, it shows the marker bands for *n*-C_9_ T_i_ solid phase at 1449 cm^–1^, 1463 cm^–1^, 1482 cm^–1^, 2715 cm^–1^, and 2891 cm^–1^ and for the *n*-C_10_ solid phase at 228, 1008 cm^–1^, 1473 cm^–1^, 1513 cm^–1^, 2861 cm^–1^, 2882 cm^–1^, and 2955 cm^–1^. No significant changes in the Raman spectrum were detected below 221.15 K. However, at this temperature, marker bands characteristic of the *n*-C_9_ liquid phase can be already identified at 870 cm^–1^, 1075 cm^–1^, and 1161 cm^–1^. At 223.15 K, the overall spectrum resembles that of the *n*-C_9_ liquid phase. At 233.15 K, the Raman spectrum of the mixture is a superposition of those of the liquid phases of *n*-C_9_ and *n*-C_10_. For this mixture, the obtained Raman spectra provide no evidence of the polymorphic transition.

In the case of *x*_nonane_ = 0.50 sample (Fig. 10), at 193.15 K, similarly to the *x*_nonane_ = 0.68, the spectrum of the mixture corresponds to a superposition of those of the individual components. The polymorphic transition for *n*-C_9_ occurs at 215.15 K, as evidenced by a marker band at 1420 cm^–1^. At 221.15 K, weak marker bands at 260 and 870 cm^–1^ become evident, suggesting the emergence of the *n*-C_9_ liquid phase. At 224.15 K, the spectrum demonstrates a resemblance to those of the liquid phases of both compounds. The bands at 260, 868, 1074, and 1160, along with the shoulder at 1310 cm^–1^ and the overall shape of the broad band around 1450 cm^–1^, are all typical of the Raman spectra of the liquid phases. The absence of *n*-C_9_ solid-phase Raman bands was noticed at 227.15 K; however, persisting bands at 1005 cm^–1^ and 1471 cm^–1^ indicated the presence of residual *n*-C_10_ solid phase. These bands completely disappear in a temperature interval between 233.15 K and 238.15 K. At 238.15 K, the spectrum becomes similar to that obtained at room temperature.

Finally, for the *x*_nonane_ = 0.20 sample (Fig. 11), at 193.15 K, the spectrum shows the marker bands at 1448 cm^–1^, 1464 cm^–1^, 1483 cm^–1^, and 2891 cm^–1^ for the *n*-C_9_ T_i_ solid phase and at 231 cm^–1^, 1009 cm^–1^, 1176 cm^–1^, 1474 cm^–1^, 1514 cm^–1^, 2862 cm^–1^, 2883 cm^–1^, 2935 cm^–1^, 2955 cm^–1^, and 2967 cm^–1^ for the *n*-C_10_ solid phase. The spectrum exhibits a stronger contribution from the *n*-C_10_ solid phase due to its higher concentration in the mixture. The first changes in the spectrum, indicating a solid–liquid transition, are observed at 235.15 K, where the marker bands characteristic of the *n*-C_9_ and *n*-C_10_ liquid phases appear in the regions of 240–280 cm^–1^, 825–940 cm^–1^, and 1070–1100 cm^–1^, along with a shoulder at 1310 cm^–1^. At 240.15 K, almost all marker bands for the solid phase have disappeared, indicating that the mixture is nearly in the liquid state. At 248.15 K, the sample is completely in the liquid state, and the spectrum is almost equivalent to that obtained at room temperature. Similar to the *x*_nonane_ = 0.30 mixture, no polymorphic transition has been detected for this mixture using Raman spectroscopy, due to the low concentration of *n*-C_9._

The study of the binary mixtures demonstrated that polymorphism in the sample becomes considerably more challenging to detect by Raman spectroscopy upon decreasing the molar fraction of *n*-C_9_, as it could be anticipated. For example, in the binary mixture with *x*_nonane_ = 0.95, the polymorphic transition was distinctly clear. However, at *x*_nonane_ = 0.80, this transition became significantly harder to discern. The difficulty escalated further as the mixture composition approached that of pure *n*-C_10_. Notably, for mixtures with compositions *x*_nonane_ = 0.30 and *x*_nonane_ = 0.20, no clear evidence of a polymorphic transition was observed by Raman spectroscopy, even though DSC results revealed a small, yet significant peak, confirming its occurrence. Additionally, for the *x*_nonane_ = 0.10 sample, no polymorphic transition is detected using both DSC and Raman techniques. Similarly, HSM has also encountered difficulties to identify these polymorphic events for the samples with a larger percentage of *n*-C_10_. Overall, for the binary mixtures studied, visualizing the polymorphic transition proved to be difficult, particularly at higher concentrations of *n*-C_10_.

### Solid–Liquid Phase Diagram

**Fig. 12:**
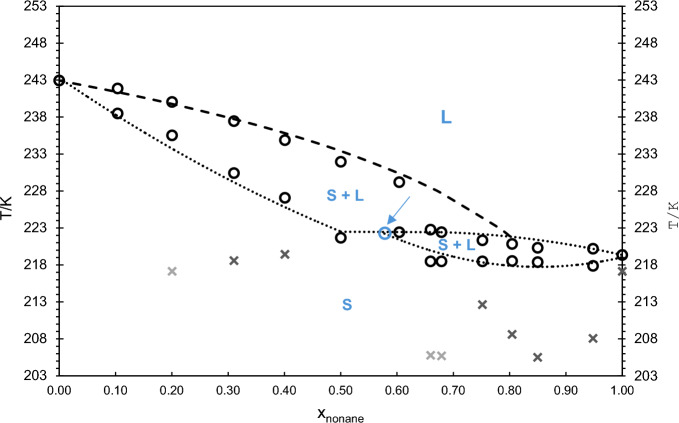
Proposed binary solid–liquid phase diagram for the *n*-C_9_ and *n*-C_10_ mixture. **·····** *liquidus* and *solidus* lines, **– – – –** fitted *liquidus* line, **O** experimental *liquidus* and *solidus* data points; × solid–solid transitions for pure *n*-C_9_ detected by all the three techniques, × solid–solid transitions for pure *n*-C_9_ detected by DSC only;  possible peritectic point according to the experimental results

The construction of the solid–liquid binary phase diagram, shown in Fig. 12, was based on the DSC results, significantly supported by Raman spectroscopy. The complete interpretation of DSC results alone is frequently very difficult, as it is often not possible to assign the observed peaks to a specific thermal event. Therefore, complementary methods, for example, HSM and Raman spectroscopy, are generally required. In this study, Raman spectroscopy was indeed crucial for the identification of three different thermal signatures for the binary mixtures.

As a matter of fact, one type of binary mixtures exhibits two peaks in the thermograms, one peak corresponding to the polymorphic (solid–solid) transition and the other one to the phase transition from solid to liquid. The mixtures that have shown this behavior are as follows: *x*_nonane_ = 0.95, 0.85, 0.75, 0.40, 0.30, and 0.20. On the other hand, the *x*_nonane_ = 0.50 and *x*_nonane_ = 0.60 samples also present two peaks in the thermograms, but both corresponding to the solid–liquid phase transition, with the first peak corresponding to the beginning of the transition and the second one to its end. Finally, a third type of mixtures show only one peak in the thermogram. In these cases, the polymorphic transition could not be confirmed either using DSC or Raman techniques. The mixtures that evidenced this behavior are as follows: *x*_nonane_ = 0.68, 0.66, and 0.10.

For the *x*_nonane_ = 0.50 and *x*_nonane_ = 0.60 samples, the thermograms were not clear enough to eliminate the possibility of a polymorphic transition. This was motivated because (i) upon heating the sample, a subtle curvature is present before the solid–liquid phase transition peak and (ii) upon cooling the sample, a small, isolated peak was observed, in addition to the solid–liquid transition. However, following the Raman results, which did not confirm the occurrence of polymorphism, it was decided to not include these two points polymorphic transition in the binary phase diagram, as their confirmation remains uncertain.

In the case of the binary mixture *x*_nonane_ = 0.10, the thermogram does not show any type of evidence of a polymorphic transition, the results being very similar to those obtained for the pure component, *n*-C_10_.

Taking into account the above-discussed results, the construction of the solid–liquid phase diagram, shown in Fig. 12, was based on the onset temperatures, *T*_*onset*_, for the *solidus* line, and on the maximum temperatures, *T*_*max*_, for the *liquidus* line. In this way, it is intended to ensure that, for the three different types of behavior discussed above, the *solidus* line temperature corresponds to the beginning of the solid–liquid transition, regardless of the number of peaks in the thermograms.

The polymorphic transitions are signaled in the phase diagram (Fig. 12) for the mixtures that have clearly evidenced them. For this binary system, certain mixtures did not exhibit polymorphic transitions, while others, such as those with molar fractions of x_nonane_ = 0.20, 0.66, and 0.68, displayed polymorphic transitions detectable only by the DSC technique. Additionally, there were also some binary mixtures that evidenced polymorphic transitions that were detected by all the three experimental techniques (DSC, HSM, and Raman spectroscopy). In light of these variations, the polymorphic transitions are differentiated in Fig. 12, as specified in the figure caption.

The experimental temperature values, *T*_*onset*_ and *T*_*max*_, obtained by DSC for both types of phase transitions (solid–solid and solid–liquid), are given in Tables 2 and S1 for all studied compositions of the binary mixtures and their pure components. Particularly, the *solidus* and *liquidus* temperatures represented in the binary phase diagram are given in Table S2 in the Supplementary Information.

The results for the system under study evidence the existence of a peritectic phase diagram, with a peritectic composition around *x*_nonane_ = 0.60 (indicated by the blue arrow and circle). Raman spectroscopy further revealed that the system is partially isomorphous, as both components have some solubility in the solid phase, although limited. In the Raman spectra, marker bands for liquid *n*-C_9_ and *n*-C_10_ appear simultaneously at the onset of the solid–liquid transition. This indicates that the solid phase that is melting and is formed by the two components, *n*-C_9_ and *n*-C_10_.

The solubility limits in the solid state could not be determined, because Raman spectroscopy does not provide quantitative compositional analysis. The *liquidus* line on the left side of the diagram (indicated by **– – – –**) was fitted using the freezing point depression curve [14], which has been applied in our previous studies on eutectic systems [3, 4]. Despite considerable variation in the composition of the solid phase, the equation shown, for instance in Denbigh [14], provided a good fit to our experimental data.

This work is part of a broader study on phase equilibria of mixtures of *n*-alkanes, originally aimed at evaluating their potential as PCM for low-temperature (TES) applications. The work has further led to a parallel investigation into odd–even effects in binary *n*-alkane mixtures and their implications for phase equilibrium behavior. In particular, the binary system composed of *n*-C_9_​(odd) and *n*-C_10_​(even) confirms findings of previous studies, namely, those reported in our previous article [2] regarding the odd–even effects in *n*-alkane mixtures phase behavior and the interpretations made therein. In particular, the solid–liquid phase diagram of this system confirms a peritectic behavior, consistent with previously observed trends [2]. However, as a peritectic system, this mixture lacks the ideal properties for TES applications. In practice, peritectic systems are associated with metastability issues and phase separation, which render them less suitable for use in TES applications compared to the other system types, as it has been previously stated [10].

Furthermore, based on the enthalpy of fusion values, Δ_*fus*_*H*, presented in Tables 2 and S1, it can be inferred that this system is not the most appropriate for TES in terms of its storage capacity. The highest enthalpy value, approximately 146.1 J·g^–1^, corresponds to the mixture with x_nonane_ = 0.20, while the enthalpy of fusion for the mixture nearest to the peritectic point, with x_nonane_ = 0.60, is 78.1 J·g^–1^. In fact, when compared to other systems previously studied by our research group, this system demonstrates a slight reduction in energy storage capacity regarding its intended application as a PCM in TES.

Overall, this study not only concludes the series of phase equilibrium studies on *n*-alkanes at low temperatures of this research program but also provides an assessment of the suitability of the specific studied system for TES applications.

## Conclusion

*n*-alkanes have been widely studied for TES applications as PCM, owing to their advantageous properties compared to other systems, as previously reported [2]. However, the limited data available in the literature for low-temperature *n*-alkane systems made us to initiate a series of phase equilibrium studies on binary systems of linear alkanes to evaluate their potential as PCM for TES applications. The present work is a further contribution to that series of binary *n*-alkane systems, involving an odd (*n*-C_9_) and an even (*n*-C_10_) carbon atoms.

The binary phase diagram obtained for this system was constructed using DSC and Raman spectroscopy, complemented by hot-stage polarization microscopy to enhance the experimental dataset. From the DSC analysis, the temperatures and enthalpies of fusion were determined for twelve binary mixtures with different molar compositions. Raman spectroscopy played an essential role in complementing the DSC results and assigning thermal events to specific DSC peaks. The system exhibited a peritectic point at *x*_nonane_ = 0.60, with a peritectic temperature, *T*_*fus*_, of 222.41 K.

Although this binary system demonstrates some potential as a PCM for TES applications in terms of its temperature of fusion, its phase change characteristics, namely, peritectic behavior and enthalpy of fusion render it less favorable in comparison to other systems previously studied [2–4].

To the best of the authors’ knowledge, the solid–liquid phase diagram for the *n*-C_9_​ + *n*-C_10_​ system has not been previously reported. Hence, the present findings not only provide insights into the potential of this system for low-temperature energy storage but also significantly enrich the body of fundamental phase equilibrium research on *n*-alkanes at sub-zero temperatures, providing novel data that contribute to the advancement of the field.

## Supplementary Information

Below is the link to the electronic supplementary material.

Supplementary file1 (DOCX 5553 KB)

## Data Availability

Data will be made available on request. No datasets were generated or analyzed during the current study.
