# Supplementary material for: Phase Equilibrium of n-Nonane + n-Decane for Low-Temperature Thermal Energy Storage: Insights into Odd–Even Effects
Source: Int J Thermophys. 2025 Mar 6;46(4):60. doi: 10.1007/s10765-025-03531-7 (PMC11882680; doi:10.1007/s10765-025-03531-7)
Supplement: Supplementary file 1 — Supplementary file1 (DOCX 5553 KB) [file 10765_2025_3531_MOESM1_ESM.docx]

**Supplementary Information for**

**Phase Equilibrium of *n*-Nonane + *n*-Decane for Low–Temperature Thermal Energy Storage; Insights into Odd–Even Effects**

Maria C.M. Sequeira^1^, Timur Nikitin^2^, Fernando J.P. Caetano^3,4*^, Hermínio P. Diogo^1*^, João M.N.A. Fareleira^1^, Rui Fausto^2,5^

^1^ Centro de Química Estrutural, Institute of Molecular Sciences, Departamento de Engenharia Química, Instituto Superior Técnico, Universidade de Lisboa, Av. Rovisco Pais, 1049-001 Lisboa, Portugal

^2^ CQC-IMS, Departamento de Química, Universidade de Coimbra, Rua Larga, 3004-535 Coimbra, Portugal

^3^ Departamento de Ciências e Tecnologia, Universidade Aberta, Rua da Escola Politécnica, 147, 1269-001 Lisboa, Portugal

^4^ Centro de Química Estrutural, Institute of Molecular Sciences, Instituto Superior Técnico, Universidade de Lisboa, Av. Rovisco Pais, 1049-001 Lisboa, Portugal

^5^ ERA-Chair Spectroscopy@IKU, Faculty of Sciences and Letters, Dept. of Physics, Istanbul Kultur Univ., Ataköy Campus, Bakirköy 34156, Istanbul, Turkey

*Corresponding Author: fernando.caetano@uab.pt; hdiogo@tecnico.ulisboa.pt

**Contents**

**Section S1: DSC data…………………………………………………………..............................................3**

- **Fig. S1** DSC heating curves of C_9_/C_10_ binary mixtures, with *n*-C_9_ molar fraction *x*_nonane_, β = 5 K·min^-1^ (exo up).
- **Table S1** DSC data for pure *n-*C_9_ [1], pure *n*-C_10_ [2] and for their binary mixtures, including the onset temperatures, *T_onset_*, maximum peak temperatures, *T_max_*, and the corresponding enthalpies of fusion, Δ_fus_*H*, at atmospheric pressure, 0.1 MPa. The scanning rate was *β* =2 K·min^–1^ for pure *n-*C_9_ and *β* = 5 K·min^–1^ for *n-*C_10_ and for the binary mixtures [1,2].

**Section S2: HSM data………………………………………………………………………….…………..…...4**

- **Fig. S2** HSM results for *n*-C_9_ [1]
- **Fig. S3** HSM results for the binary mixture with molar fraction *x*_nonane_ = 0.95
- **Fig. S4** HSM results for the binary mixture with molar fraction *x*_nonane_ = 0.80
- **Fig. S5** HSM results for the binary mixture with molar fraction *x*_nonane_ = 0.75
- **Fig. S6** HSM results for the binary mixture with molar fraction *x*_nonane_ = 0.60
- **Fig. S7** HSM results for the binary mixture with molar fraction *x*_nonane_ = 0.40

**Section S3: Raman Spectroscopy data………………………………………….………….……………...6**

- **Fig. S8** Temperature-variation Raman spectra for the pure *n*-C_9_ [1]
- **Fig. S9** Temperature-variation Raman spectra for the binary mixture *x*_nonane_ = 0.95
- **Fig. S10** Temperature-variation Raman spectra for the binary mixture *x*_nonane_ = 0.75
- **Fig. S10** Temperature-variation Raman spectra for the binary mixture *x*_nonane_ = 0.66
- **Fig. S11** Temperature-variation Raman spectra for the binary mixture *x*_nonane_ = 0.30
- **Fig. S12** Temperature-variation Raman spectra for the binary mixture *x*_nonane_ = 0.10

**Section S4: Statistical Parameters and data……………………………………………..…………...…...9**

**Section S5: Data for the Binary Phase Diagram Construction……………………………………..…10**

# **S1. DSC Data**

**
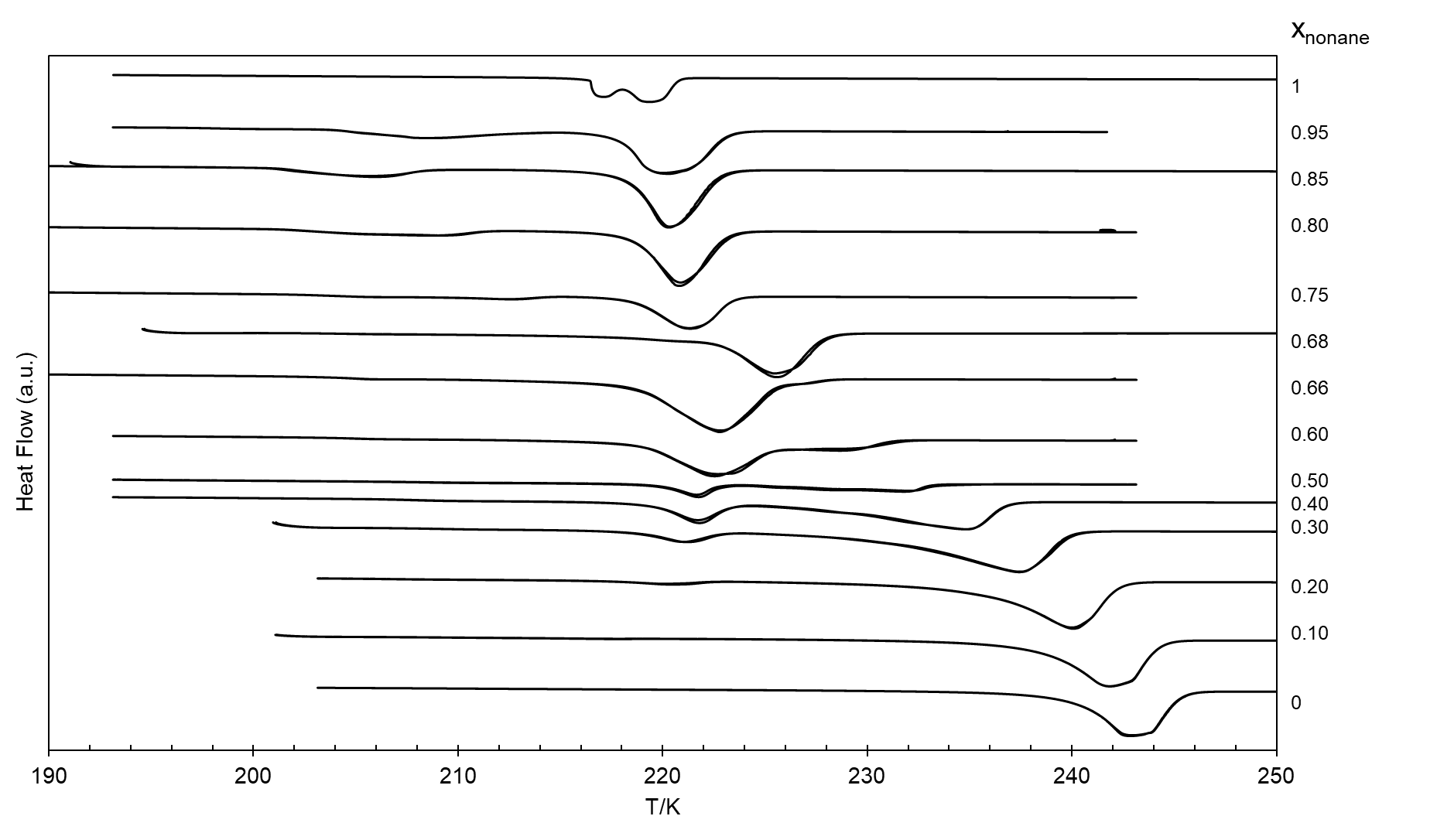
**

**Fig. S1** DSC heating curves of pure compounds *n*-C_9_ [1] and *n*-C_10_ [2] and for their binary mixtures, with signaled *n*-C_9_ molar fraction *x*_nonane_. The scanning rate was *β* = 2 K·min^-1^ for pure *n*-C_9_ [1] and *β* = 5 K·min^-1^ for pure *n*-C_10_ [2] and the C_9_/C_10_ binary mixtures (exo up). Two consecutive heating runs are presented for each composition.

**Table S1** DSC data for pure *n-*C_9_ [1], pure *n*-C_10_ [2] and for their binary mixtures, including the onset temperatures, *T_onset_*, maximum peak temperatures, *T_max_*, and the corresponding enthalpies of fusion, Δ_fus_*H*, at atmospheric pressure, 0.1 MPa. The scanning rate was *β* =2 K·min^–1^ for pure *n-*C_9_ and *β* = 5 K·min^–1^ for *n-*C_10_ and for the binary mixtures [1,2].

| **x_nonane_** | **DSC 1^st^ peak** | | | **DSC 2^nd^ peak** | | |
| --- | --- | --- | --- | --- | --- | --- |
|  | ***T*_onset_ /K** | ***T*_max_ /K** | **Δ_fus_*H* /J·g^–1^** | ***T*_onset_ /K** | ***T*_max_ /K** | **Δ_fus_*H* /J·g^–1^** |
| 1^a)^ | 216.46 | 217.12 | 3.8 | 218.28 | 219.34 | 7.4 |
| 0.9481 | 203.15 | 208.06 | 25.8 | 217.87 | 220.18 | 107.7 |
| 0.8499 | 200.82 | 205.50 | 20.9 | 218.37 | 220.31 | 103.6 |
| 0.8044 | 201.23 | 208.60 | 22.0 | 218.53 | 220.83 | 104.7 |
| 0.7516 | 209.46 | 212.63 | 7.1 | 218.47 | 221.32 | 107.8 |
| 0.6790 | 218.48 | 222.42 | 116.3 | – | – | – |
| 0.6596 | 218.48 | 222.77 | 107.9 | – | – | – |
| 0.6040 | 218.82^b)^ | 222.41 | 78.1^b)^ | –^c)^ | 229.18 | –^c)^ |
| 0.5002 | 219.81 | 221.67 | 38.7 | 224.67 | 231.95 | 54.7 |
| 0.4008 | 219.43 | 221.69 | 27.9 | 227.07 | 234.87 | 85.9 |
| 0.3104 | 218.58 | 220.99 | 13.9 | 230.41 | 237.44 | 117.7 |
| 0.2005 | 217.14 | 220.11 | 6.2 | 235.51 | 240.03 | 146.1 |
| 0.1038 | 238.48 | 241.86 | 165 | – | – | – |
| 0^a)^ | 240.19 | 242.93 | 169.4 | – | – | – |

Expanded uncertainties for a 95% confidence level (*k* = 2): *U*(*x*) = 0.00007; *U*(*T*) = 0.25 K; *U*(Δ_fus_*H)* = 8.1 J·g^–1^ (see Supp. Information – S4).

^a)^ Values previously published [1,2]; ^b)^ Overall *T_onset_* and Δ_fus_*H* value for the two overlapped peaks; ^c)^ Not possible to obtain from the integration.

# **S2. HSM Data**


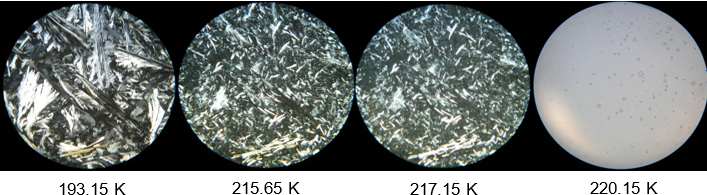


**Fig. S2** HSM images of *n*-C_9_ acquired on heating in the temperature range between 193.15 K and 220.15 K, employing a magnification of 250× [1].

**
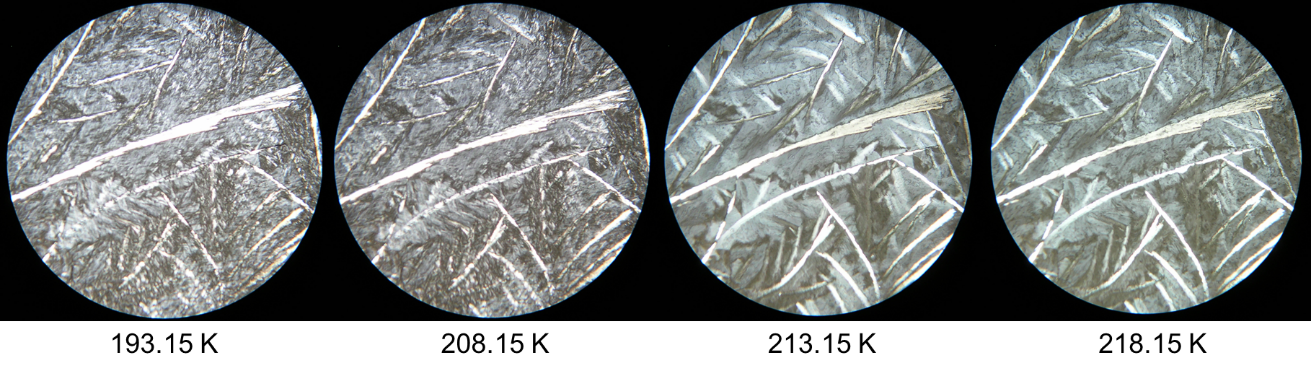
**

**Fig. S3** HSM images of the binary mixture with molar fraction *x*_nonane_ = 0.95 acquired on heating in the temperature range between 193.15 K and 218.15 K, employing a magnification of 250×.


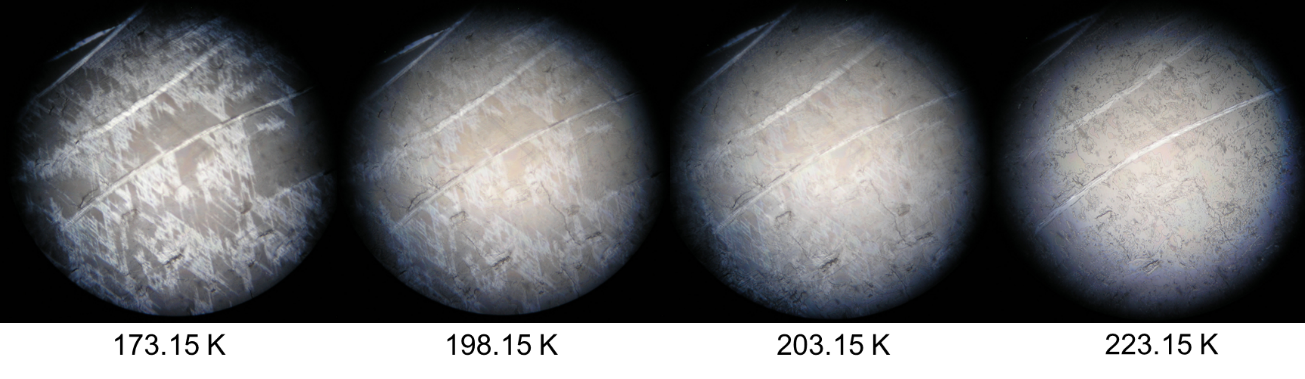


**Fig. S4** HSM images of the binary mixture with molar fraction *x*_nonane_ = 0.85 acquired on heating in the temperature range between 173.15 K and 223.15 K, employing a magnification of 250×.


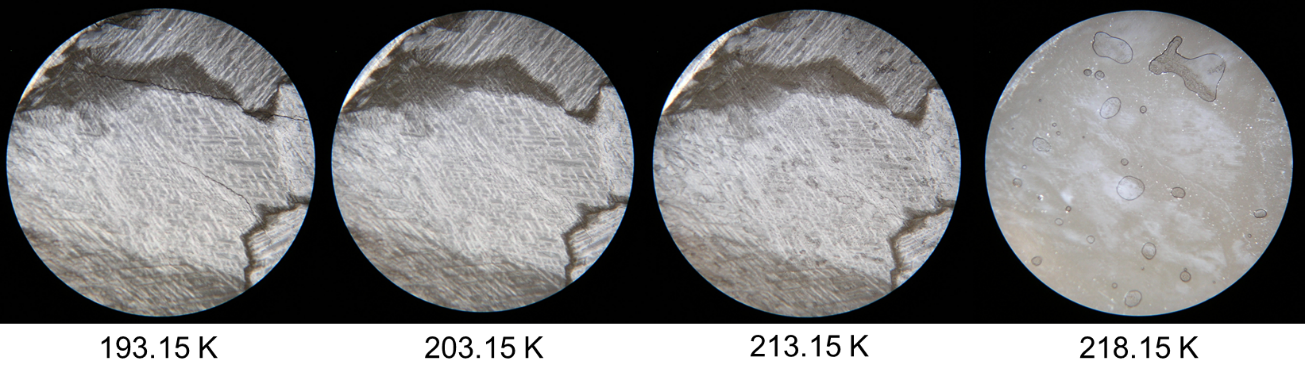


**Fig. S5** HSM images of the binary mixture with molar fraction *x*_nonane_ = 0.75 acquired on heating in the temperature range between 193.15 K and 218.15 K, employing a magnification of 250×.

**
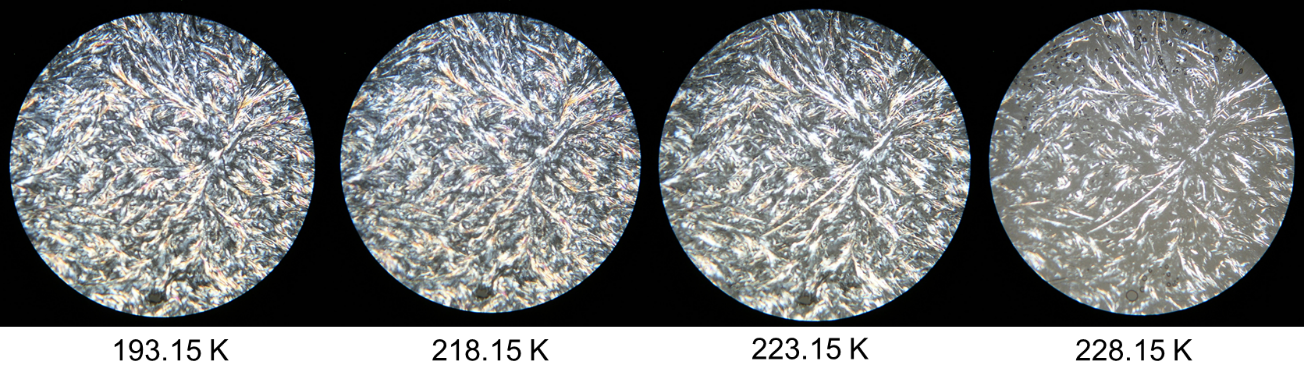
**

**Fig. S6** HSM images of the binary mixture with molar fraction *x*_nonane_ = 0.60 acquired on heating in the temperature range between 193.15 K and 228.15 K, employing a magnification of 250×.

**
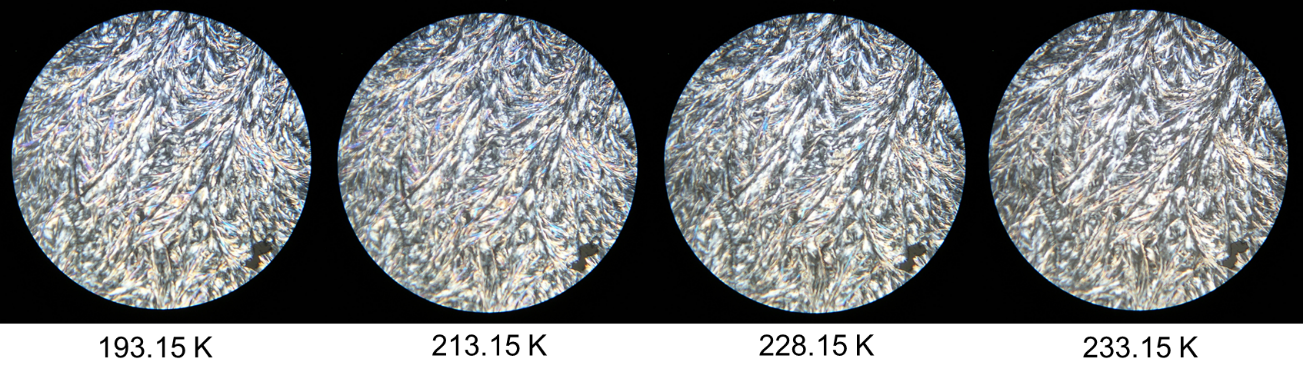
**

**Fig. S7** HSM images of the binary mixture with molar fraction *x*_nonane_ = 0.40 acquired on heating in the temperature range between 193.15 K and 233.15 K, employing a magnification of 250×.

# **S3. Raman Spectroscopy Data**

- Binary mixture *x*_nonane_ = 0.95:

At the highest concentration of *n*-C_9_ in the mixture, its spectrum at 193.15 K is equivalent to that of the T_i_ solid phase of *n*-C_9_. At 208.15 K, the marker bands at 2870, 2890, and 2960 cm^–1^ begin their transformation to shoulders, and the appearance of a very weak band/shoulder at 1421 cm^–1^ can be observed, which may indicate the onset of the polymorphic transition. When the temperature reached 211.15 K, the three high-energy shoulders become less visible, indicating a further transformation to the *n*-C_9_ R_I_ solid phase. At 214.15 K, the band/shoulder at 1421 cm^–1^, also related to the *n*-C_9_ R_I_ solid phase, becomes more pronounced. The transformation to the liquid phase starts at temperatures between 217 and 220.15 K. By 220.15 K, the sample must be nearly liquid, since the spectrum closely resembles that of the liquid phase. The most prominent bands for the *n*-C_9_ liquid phase can be seen at 264, 871, 889, 1075, 1161, 1456, 2872, and 2959 cm^–1^. At 95C_9_:5C_10_ composition ratio, it was challenging to identify the marker bands for *n*-C_10_ in both solid and liquid phases, which is justified by the composition of this binary mixture, where *n*-C_10_ is almost vestigial. At 233.15 K, the sample is completely liquid.


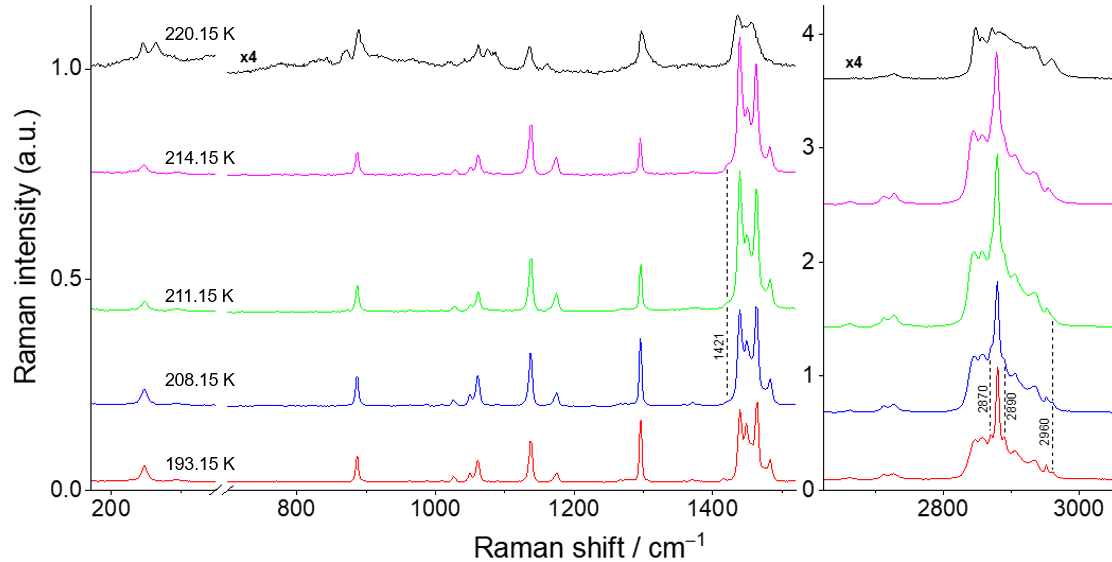


**Fig. S8** Temperature-variation Raman spectra for the binary mixture x_nonane_ = 0.95.

- Binary mixture *x*_nonane_ = 0.75:

At 193.15 K, the spectrum evidences the marker bands for the *n*-C_9_ T_i_ solid phase at 1450, 1464, and 1483 cm^–1^ and the marker bands for *n*-C_10_ solid phase at 230, 1009, a shoulder at 1474, 1514, 2863, 2882, 2936, and 2955 cm^–1^. Similarly to the 80C_9_:20C_10_ mixture, at 208.15 K it is possible to identify a very subtle broad band at 1421 cm^–1^ – a marker band for the *n*-C_9_ R_I_ solid phase, which continues to grow reaching its highest intensity at 216.15 K and remaining constant up to 222.15 K. The transformation to the liquid phase occurs between 222.15 and 223.15 K, which correlates very well with the DSC peak observed at 222.15 K. At 223.15 K, the spectrum resembles that of the liquid phase. Especially the broad band at about 1450 cm^–1^ and the group of bands between 2850 and 2975 cm^–1^. At 233.15 K, the spectrum is already equivalent to the one obtained at room temperature.


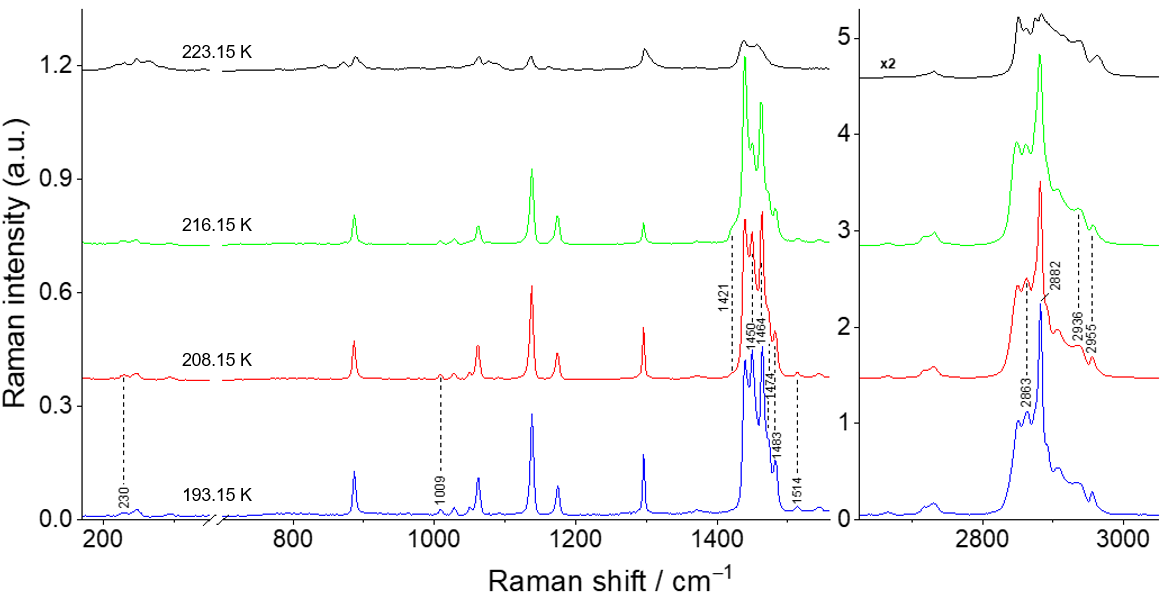


**Fig. S9** Temperature-variation Raman spectra for the binary mixture *x*_nonane_ = 0.75.

- Binary mixture *x*_nonane_ = 0.66:

At 193.15 K, the spectrum of the mixture shows the marker bands of *n*-C_9_ T_i_ solid phase at 1449, 1463, 1482, 1543, 2715, 2881, and 2889 cm^–1^ and of the *n*-C_10_ solid phase at 1008, 1472, 1513, 2849, 2862, 2881, and 2954 cm^–1^. At 209.15 K, it is possible to notice an appearance of a small shoulder at 1420 cm^–1^, which may indicate the beginning of the polymorphic transition to the solid R_I_ phase. Interestingly, the DSC results show only a weak peak around 206.15 K. Upon further heating to 221.15 K, the spectrum of the mixture becomes more similar to that of the solid R_I_ phase, which is evidenced by the growth of the band 1420 cm^–1^, and slight redshift of the following bands: 2849 to 2846, 2861 to 2860, and 2881 to 2880 cm^–1^. At 224.15 K, the spectrum of the mixture transforms to that very similar of the mixture of liquid phases of *n*-C_9_ and *n*-C_10_, with some residual peaks related to the solid phases. At 230.15 K, the marker bands for solid phases are no longer visible and only the marker bands for liquid phases are identifiable in the spectrum of the mixture. At 233.15 K, the spectrum is equivalent to the one obtained at room temperature.


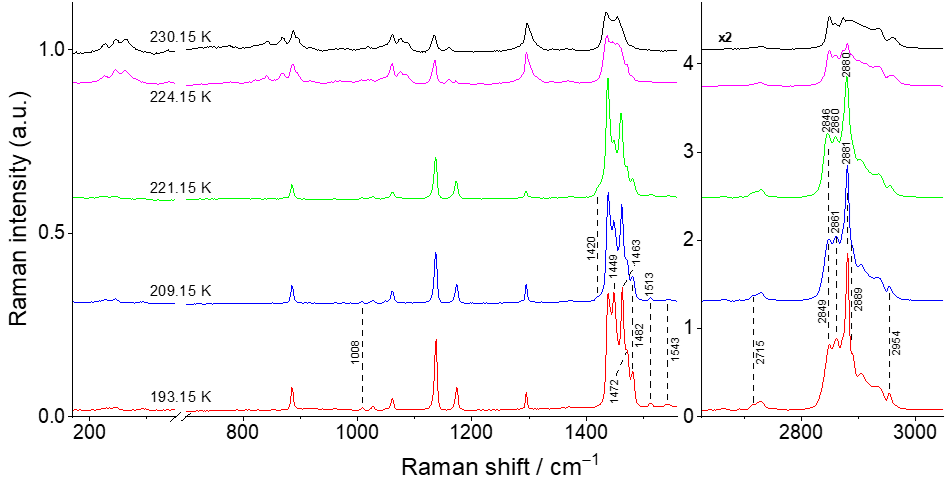


**Fig. S10** Temperature-variation Raman spectra for the binary mixture x_nonane_ = 0.66.

- Binary mixture *x*_nonane_ = 0.30:

At 193.15 K, the spectrum of the mixture displays all the characteristic marker bands of both the *n*-C_9_ T_i_ and *n*-C_10_ solid phases, with the latter being more prominent due to its higher concentration. The Raman spectra remained consistent upon heating until the temperature reached 222.15 K, at which point weak bands characteristic of the liquid phases began to emerge at 866, 1072, and 1158 cm^–1^. As the temperature increased to 234.15 K, additional marker bands for the liquid phase appeared, including those at 839, 892, 914, 1015, 2871, and 2959 cm^–1^. By 237.15 K and above, the spectrum of the mixture matched the one recorded at room temperature. Notably, no evidence of a polymorphic transition was observed in this mixture, likely attributable to the lower concentration of *n*-C_10_.


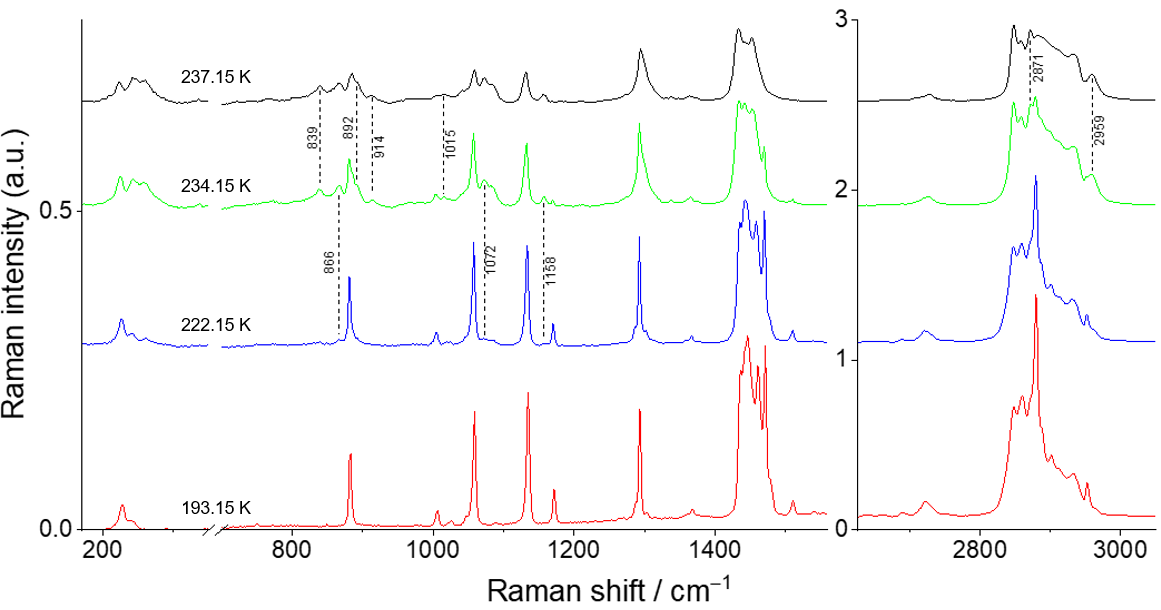


**Fig. S11** Temperature-variation Raman spectra for the binary mixture *x*_nonane_ = 0.30.

- Binary mixture *x*_nonane_ = 0.10:

At a temperature of 193.15 K, because of the low concentration of *n*-C_9_ in the mixture, its spectrum mirrors that of the *n*-C_10_ solid, with no distinguishable bands of *n*-C_9_ observed. Transition to the liquid phase begins between 240.15 K and 243.15 K, which is also in accordance with the DSC results. At –33 ºC, the spectrum is already very close to that of the liquid phase of *n*-C_10_. At 248.15 K, the spectrum is equivalent to the one obtained at room temperature.


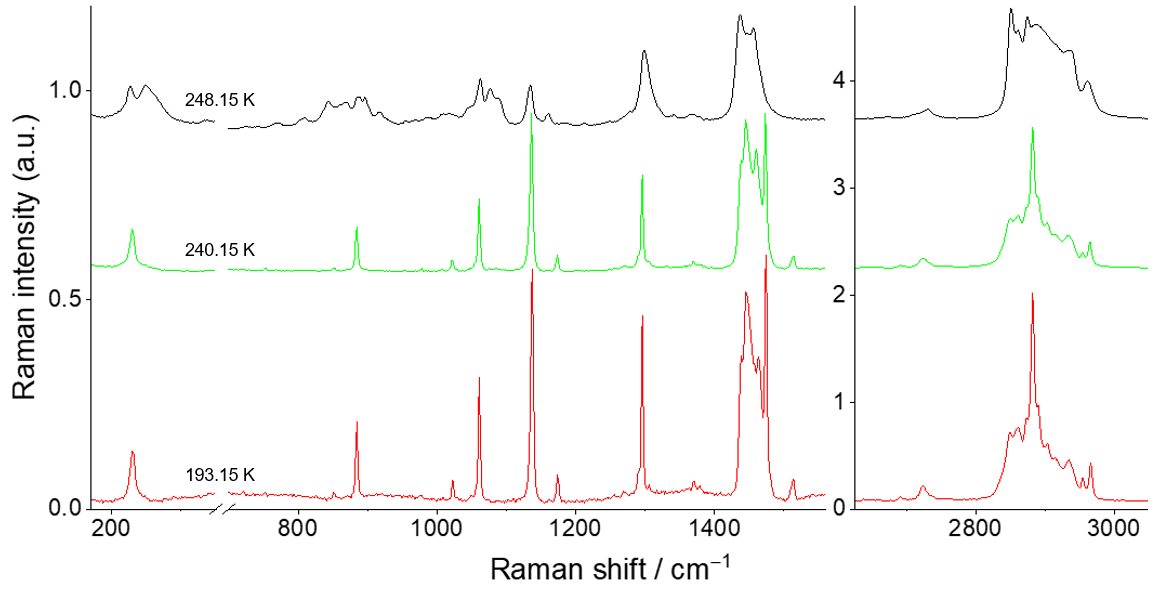


**Fig. S12** Temperature-variation Raman spectra for the binary mixture *x*_nonane_ = 0.10.

# **S4. Statistical Parameters and Data**

The statistical parameter used to calculate the expanded uncertainty of the temperatures and enthalpies of fusion in this work is the same as that used in the previous ones [1,2] and is defined by Eq. (S1), in particular, the standard deviation, hereby designated as *σ*.

(S1)

$$\sigma=\left[ \frac{{\sum(X_{i}-\bar{X})}^{2}}{N-1} \right]^{1/2}$$

where *N* is the total number of experimental data points, the subscript *i* stands for the *i-th* experimental point, respectively, and *X* stands for temperature or enthalpy values.

The expanded uncertainty *U* is calculated according to Eq. (S2).

(S2)

$$U=2\sigma$$

The estimation of the expanded uncertainties for temperatures (*T_onset_* and *T_max_*) and the enthalpy of fusion (Δ_fus_*H*) is based on five independent measurements using the experimental procedure described in Section 2.2.1. The five prepared samples correspond to the binary mixture with composition *x*_nonane_ = 0.80 since it only presents a one very well–defined DSC peak. This estimation is assumed to be valid for all the experimental measurements.

The estimation of the expanded uncertainties for molar fractions (*x*_nonane_ and *x*_decane_) is based on the error propagation analysis considering the weighing of the samples and the uncertainty of the molecular weights of these compounds. For the estimation of the uncertainty of molecular weights, the NIST Technical Note 1900 [2,3] and the IUPAC Technical Report published in 2022 were used [4].

# **S5. Data for the Binary Phase Diagram Construction**

As explained in the article, the construction of the binary phase diagram is a result of the three experimental techniques used: DSC, HSM and Raman spectroscopy. In fact, the diagram present is mostly a result of the DSC and Raman techniques. With these two techniques it is possible to obtain the temperatures and enthalpies of each thermal event and then, using Raman spectroscopy, understand and identify these thermal events.

After the analysis of the Raman experimental results, it became clear that for the binary mixtures presenting polymorphism and two DSC peaks, e.g. *x*_nonane_=0.95, 0.85, 0.75, 0.40, 0.30 and 0.20, and this event the small first peaks observed in the DSC thermograms could be assigned to the polymorphic transition. Consequently, the phase change from solid to liquid would only happen in the second DSC peak. This means that the *T_onset_* corresponds, in these cases, to the beginning of the phase transition and the *T_max_* to the end of this transition.

For the binary mixtures *x*_nonane_=0.50 and *x*_nonane_=0.60 that also present two DSC peaks, the Raman spectroscopy confirmed that these peaks corresponded only to the phase transition from solid to liquid. Consequently, in these cases the beginning of the phase transition corresponds to the *T_max_* of the first peak and the end of this thermal event corresponds to the *T_max_* of the second peak.

Finally, the mixtures with only one DSC peak, as *x*_nonane_=0.68, 0.66 and 0.10, after being analyzed by Raman spectroscopy, enabled the confirmation of the beginning and the end of the solid–liquid transition. Once again, the *T_onset_* corresponds to the beginning of the melting process and the *T_max_* to the end of it.

Following this, Table S2 shows the temperature values used for the binary phase construction, depicted from the DSC results and corroborated by Raman spectroscopy.

**Table S2** Temperature values (K) used for the construction of the *liquidus* and *solidus* lines of the binary phase diagram.

| ***x*_nonane_** | ***T_poli_*/K** | ***T_Solidus_* /K** | ***T_Liquidus_*/K** |
| --- | --- | --- | --- |
| 1^a)^ | 217.12 | - | 219.34 |
| 0.9481 | 208.06 | 217.87 | 220.18 |
| 0.8499 | 205.50 | 218.37 | 220.31 |
| 0.8044 | 208.60 | 218.53 | 220.83 |
| 0.7516 | 212.63 | 218.47 | 221.32 |
| 0.6790 | 205.70^b)^ | 218.48 | 222.42 |
| 0.6596 | 205.78^b)^ | 218.48 | 222.77 |
| 0.6040 | - | 222.41 | 229.18 |
| 0.5002 | - | 221.67 | 231.95 |
| 0.4008 | 219.43 | 227.07 | 234.87 |
| 0.3104 | 218.58 | 230.41 | 237.44 |
| 0.2005 | 217.14 | 235.51 | 240.03 |
| 0.1038 | - | 238.48 | 241.86 |
| ^a)^ | - | - | 242.93 |

^a)^ Values previously published [1,2]; ^b)^ Values depicted from DSC thermogram; the integration of the peak is not possible since it is very subtle, but the temperature value for the polymorphic event was confirmed through Raman spectroscopy.

**References**

[1] M. C. M. Sequeira, T. Nikitin, F. J. P. Caetano, H. P. Diogo, J. M. N. A. Fareleira, R. Fausto, Solid – Liquid Phase Equilibrium of the *n* ‑ Nonane + *n* ‑ Undecane System for Low ‑ Temperature Thermal Energy Storage, Int. J. Thermophys. **45**, 117 (2024). https://doi.org/10.1007/s10765-024-03411-6.

[2] M. C. M. Sequeira, B. A. Nogueira, F. J. P. Caetano, H. P. Diogo, J. M. N. A. Fareleira, R. Fausto, Solid–Liquid Phase Equilibrium: Alkane Systems for Low-Temperature Energy Storage, Int. J. Thermophys. **45**, 28 (2024). https://doi.org/10.1007/s10765-023-03317-9.

[3] A. Possolo, Simple Guide for Evaluating and Expressing the Uncertainty of NIST Measurement Results NIST Technical Note 1900, Natl. Inst. Stand. Technol. **1297**, 1–20 (2015). http://dx.doi.org/10.6028/NIST.TN.1900.

[4] T. Prohaska, J. Irrgeher , J. Benefield , J. K. Böhlke , L. A. Chesson , T. B. Coplen , T. Ding, P. J. H. Dunn, M. Gröning, N. E. Holden, H. A. J. Meijer, H. Moossen, A. Possolo, Y. Takahashi, J. Vogl, T. Walczyk, J. Wang, M. E. Wieser, S. Yoneda, X. Zhu, Juris Meija, Standard atomic weights of the elements 2021 (IUPAC Technical Report). Pure Appl. Chem. **94**, 573–600 (2022). https:// doi.org/ 10.1515/pac-2019-0603.
